# Integrating transcriptomics and metabolomics to elucidate the mechanism by which taurine protects against DOX-induced depression

**DOI:** 10.1038/s41598-023-51138-5

**Published:** 2024-02-01

**Authors:** Yanan Li, Luxi Li, Shanshan Wei, Jia Yao, Benhui Liang, Xue Chu, Lei Wang, Hui Liu, Dehua Liao, Daotong Liu, Pei Jiang

**Affiliations:** 1 College of Marine Life Sciences, Ocean University of China, No.5 Yushan Road, Qingdao, 266003 China; 2 Department of Pharmacy, Shandong Provincial Hospital Affiliated to Shandong First Medical University, Jinan, China; 3 Department of Graduate, Shandong Academy of Medical Sciences, Shandong First Medical University, Jinan, China; 4 Translational Pharmaceutical Laboratory, Jining First People’s Hospital, Shandong First Medical University, Jining, China; 5 Department of Cardiovascular Medicine, Xiangya Hospital, Central South University, Changsha, Hunan Province China; 6 Department of Pharmacy, Hunan Cancer Hospital, The Affiliated Cancer Hospital of Xiangya School of Medicine, Central South University, Changsha, China; 7 Department of Breast and Thyroid Surgery, Jining First People’s Hospital, Shandong First Medical University, Jining, China; 8 Institute of Translational Pharmacy, Jining Medical Research Academy, Jining, China

**Keywords:** Pharmacology, Toxicology, Predictive markers, Neurological disorders

## Abstract

Doxorubicin (DOX) is an effective anticancer drug with potent antitumour activity. However, the application of DOX is limited by its adverse reactions, such as depression. Taurine can alleviate depression induced by multiple factors. However, it is still unclear whether and how taurine improves DOX-induced depression. To address this question, the aim of this study was to explore the potential mechanism by which taurine protects against DOX-induced depression. Mice were randomly divided into three groups (n = 8): (1) the control group, (2) the DOX group, and (3) the DOX + taurine group. The open field test (OFT), elevated plus maze test, and forced swim test (FST) were first performed to assess the effects of DOX and taurine on the behaviour of mice. Next, a combined transcriptomic and metabolomic analysis was performed to analyse the possible antidepressive effect of taurine. Taurine pretreatment increased the total distance travelled and speed of mice in the OFT, increased the number of entries into the open arm and the time spent in the open arm, and reduced the immobility time in the FST. In addition, 179 differential genes and 51 differentially abundant metabolites were detected in the DOX + taurine group compared to the DOX group. Furthermore, differential genes and differentially abundant metabolites were found to be jointly involved in 21 pathways, which may be closely related to the antidepressant effect of taurine. Taurine alleviated DOX-induced depressive behaviour. The various pathways identified in this study, such as the serotonergic synapse and the inflammatory mediator regulation of TRP channels, may be key regulatory pathways related to depression and antidepressant effects.

## Introduction

Doxorubicin (DOX) is an anthracycline chemotherapy drug widely used in cancer treatment. DOX exerts anticancer effects by interfering with the synthesis of DNA and RNA. DOX can insert base pairs into the DNA double helix, inhibit DNA and RNA polymerases, interrupt DNA replication and RNA transcription, and thus induce cell death^1^. However, the antitumour effectiveness of DOX comes at a cost. DOX causes severe toxicity, which may lead to the development of behaviours such as depression and anxiety. Studies have shown that brain tissue is highly susceptible to the effects of DOX^2^. Although DOX cannot cross the blood‒brain barrier, DOX induces an increase in the peripheral levels of the cytokine tumour necrosis factor alpha (TNFα), which is able to cross the blood‒brain barrier, activate microglia, and promote the development of neuroinflammation, particularly in the hippocampus^3,4^. The hippocampus is a special area of the brain that is able to continuously proliferate stem cells over the course of life, and it is particularly susceptible to damage induced by DOX^5^. DOX-mediated neuroinflammation is closely related to hippocampal stem cell dysfunction, and damage to the hippocampus is considered to be an important cause of depression^6,7^. Rodent experiments indicate that after chronic exposure to clinically relevant doses of DOX, rats were impaired on two memory tasks known to rely on intact hippocampal function and exhibited disrupted neurogenesis^8^. Clinical investigation results have also shown that breast cancer patients treated with chemotherapy using DOX usually have severe negative emotions, which have an impact on the patients’ daily lives^9^. Moreover, DOX toxicity is present at multiple stages during or after treatment, resulting in persistent side effects that seriously limit the use of DOX^10^. Therefore, alleviating the toxicity of DOX has important implications for its clinical use.

Taurine is one of the most abundant amino acids in the central nervous system, and it exerts a variety of neuroprotective effects^11^. In a mouse model of ageing, taurine increases cell proliferation in the dentate gyrus through the activation of quiescent stem cells, increases the number of stem cells, and promotes neurogenesis^12^. Taurine also reduces microglial activation, thereby alleviating chronic inflammation caused by lipopolysaccharide^13^. Given the powerful effects of taurine, it may be an ideal target for the treatment of a number of disorders, including depression. Studies have shown that taurine levels are reduced in the brains of depressed rats, while taurine supplementation has antidepressant effects and the ability to change depression-related signalling cascades in the hippocampus^14^. Taurine also alleviates chronic social defeat stress-induced depression by protecting cortical neurons from dendritic spine loss and synaptic protein deficits^15^. An increasing number of studies have shown that taurine alleviates the development of depression induced by multiple factors and may become a potential therapeutic drug for depression^16^. Moreover, the neuroprotective effects of taurine may also alleviate DOX neurotoxicity^17,18^. However, it is currently unknown whether and how taurine improves DOX-induced depression. Based on the above information, the aim of the present study was to investigate whether taurine can improve DOX-induced depression, and if so, the potential mechanism underlying its protective effect against DOX-induced depression.

## Materials and methods

### Animals

**Figure 1:**
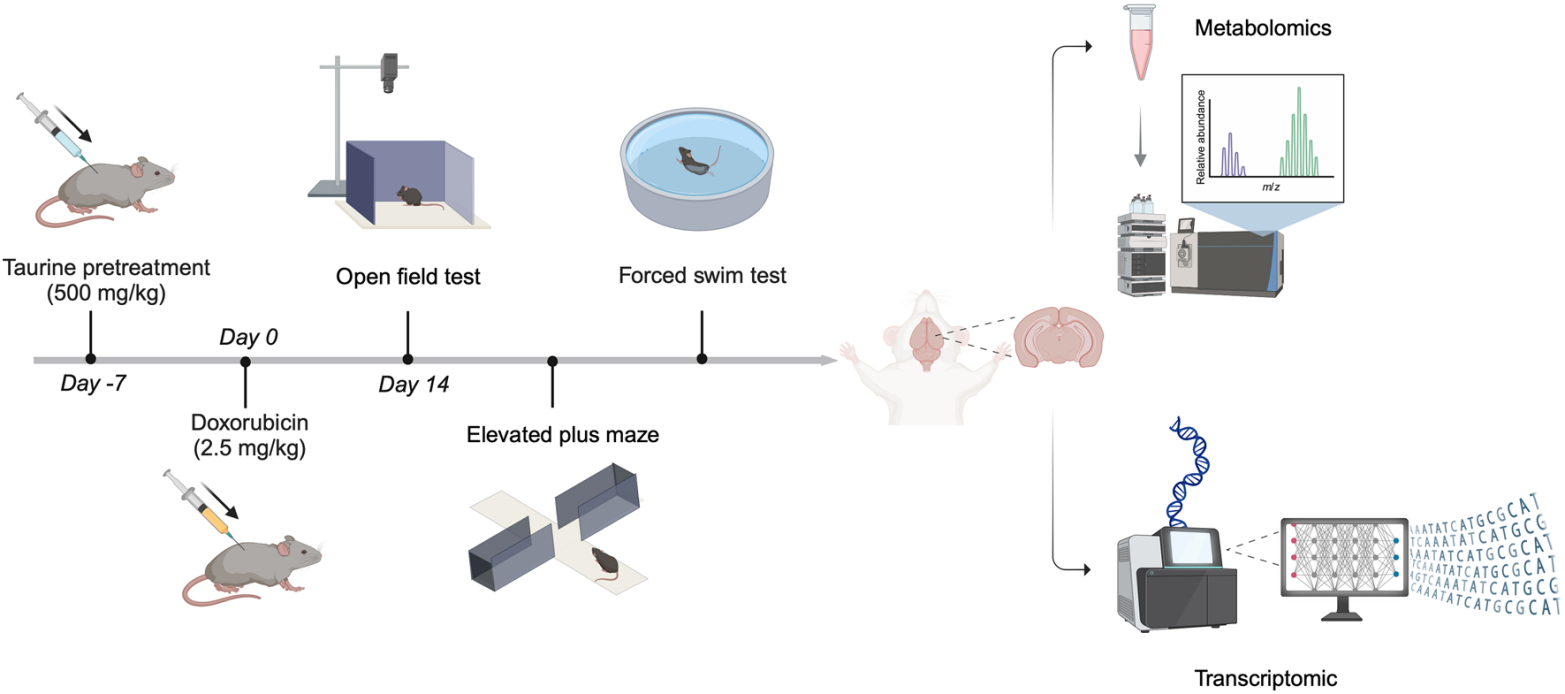
Flowchart of experimental process.

Eight-week-old C57BL/6 mice were randomly divided into three groups (n = 8): (1) the control group, (2) the DOX group, and (3) the DOX + taurine group. The untreated control group was injected with the same volume of normal saline. Mice in the DOX group received an intraperitoneal injection of DOX (2.5 mg/kg) every two days for a total of seven injections. Mice in the DOX + taurine group received the same treatment as those in the DOX group, while taurine (500 mg/kg) was injected intraperitoneally each day for one week in advance. The mice were housed at 25 °C with a 12-h light–dark cycle and had free access to water and food. Mice were then tested with the open field test (OFT), elevated plus maze test (EPM), and forced swim test (FST). The experimental process is shown in Fig. 1.

### Behavioural tests

The OFT can be used to assess the voluntary motor ability of animals. Rats were allowed to explore freely for 5 min in an automated open-air arena (100 cm long, 100 cm wide, and 40 cm high). Their trajectories were tracked, and data were collected for analysis. The total distance travelled and average speed was used as an indicator of activity level. At the end of the experiment, the rats were removed, and the entire arena box was cleaned. The experiment was repeated with a new rat after the box was dried.

The EPM test is used mainly to observe animal anxiety. The device comprises two open and two closed arms, each 50 cm long, l0 cm wide, and 20 cm high. The rats were placed in the central area and allowed to explore freely for five minutes. The number of entries into the open arm and the time spent in the open arm were indicators of anxiety levels.

The FST was performed in a transparent pool of 25 cm in height and 10 cm in diameter. The pool needed to be cleaned after swimming to ensure that the mice were in clean water for the test. Mice were placed in the water for 15 min on the first day, and the water temperature was kept at 25 °C during the experiment. After 24 h, the mice were placed in the pool again, and their immobility time was recorded for five minutes, which was used to determine the depression-like behaviour of the mice.

### Sample collection

Mice were anaesthetized by injection of sodium pentobarbital, and the hippocampal tissue was quickly removed and placed on ice. The tissue samples were stored at − 80 °C for further experiments.

### Transcriptomic analysis

Four samples from the DOX and DOX + taurine groups were used for transcriptome analysis. Total RNA was extracted using TRIzol reagent, and quantity and purity were analysed using a NanoDrop 2000 spectrophotometer (Thermo Scientific, USA). High-quality RNA samples were used to construct a sequencing library. The libraries were sequenced on an Illumina NovaSeq 6000 platform, and 150 bp paired-end reads were generated. Subsequently, clean reads were mapped to the human genome (GRCh38) using HISAT2. Gene expression levels were quantified by FPKM (fragments per kilobase of transcript per million mapped reads). Differentially expressed genes were analysed using the DESeq R package. A *P* value < 0.05 and |log2FC| ≥ 1 were used as criteria to screen differential genes. GO enrichment and KEGG pathway enrichment analyses of differentially expressed genes were performed using the clusterProfiler R package based on the hypergeometric distribution.

### Metabolomics analysis

Four samples from the DOX and DOX + taurine groups were used for the metabolomics analysis. A total of 20 mg of sample was added to 400 μL of a mixture of methanol and acetonitrile (V:V = 4:1, containing L-2-chlorophenyl alanine, 4 μg/mL). The samples were ground in a grinder (60 Hz, 2 min) and sonicated in an ice water bath for 10 min. The samples were then stationary at − 40 °C for 30 min, followed by centrifugation at 13,000 rpm for 10 min. The supernatant was dried in a freeze-concentrated centrifugal dryer. Then, a 300 μL mixture of methanol and water (V:V = 4:1) was added, and the above steps were repeated. Finally, 150 μL of the supernatant was filtered through 0.22 μm microfilters and transferred to LC vials for LC‒MS analysis.

LC‒MS/MS analyses were performed using a Dionex Ultimate 3000 RS UHPLC fitted with a Q Exactive plus quadrupole-orbitrap mass spectrometer equipped with a heated electrospray ionization (ESI) source (Thermo Fisher Scientific, Waltham, MA, USA). Chromatographic conditions were as follows: chromatographic columns, ACQUITY UPLC HSS T3 (100 mm × 2.1 mm, 1.8 um); column temperature, 45 °C; flow rate, 0.35 mL/min; injection volume, 2 µL; mobile phase A, water (containing 0.1% formic acid); and mobile phase B, acetonitrile. The ESI source was operated in both positive and negative ion modes. The mass spectrometer was operated as follows: spray voltage, 3800 V (+) and 3000 V (−); sheath gas flow rate, 35 arbitrary units; auxiliary gas flow rate, eight arbitrary units; capillary temperature, 320 °C; Aux gas heater temperature, 350 °C; and S-lens RF level, 50. The mass ranged from 100 to 1200 m/z. The resolution was set at 70,000 for the full MS scans and 17,500 for the HCD MS/MS scans. The collision energy was set at 10, 20, and 40 eV.

Original LC‒MS data were processed using Progenesis QI V2.3 software (Nonlinear, Dynamics, Newcastle, UK) for baseline filtering, peak identification, integration, retention time correction, peak alignment, and normalization. Subsequently, the data were imported into R for analysis. The model’s validity was judged using orthogonal partial least-squares discriminant analysis (OPLS-DA). A two-tailed Student’s t test was used to verify whether there were significant differences in metabolites between groups. Metabolites with variable importance (VIP) values > 1.0 in the OPLS-DA model and *P* < 0.05 in Student’s t test were considered significantly different. Then, pathway analysis of metabolites with significant differences was performed using the KEGG Pathway Library.

### Statistical analysis

The behavioural tests were processed using GraphPad Prism software 10.0, and the data are expressed as the mean ± standard error of the mean (SEM). The data were subject to normal distribution and homogeneous variance. Statistical significance was evaluated using Student’s t test or one-way ANOVA. *P* < 0.05 was used as the criterion for significance.

### Ethics approval and consent to participate

The animal study protocol was approved by the Ethics Committee of Jining First People’s Hospital (protocol no. JNRM-2022-DW-087).

## Results

### Taurine exerted a protective effect on DOX-induced depression-like behaviour

**Figure 2:**
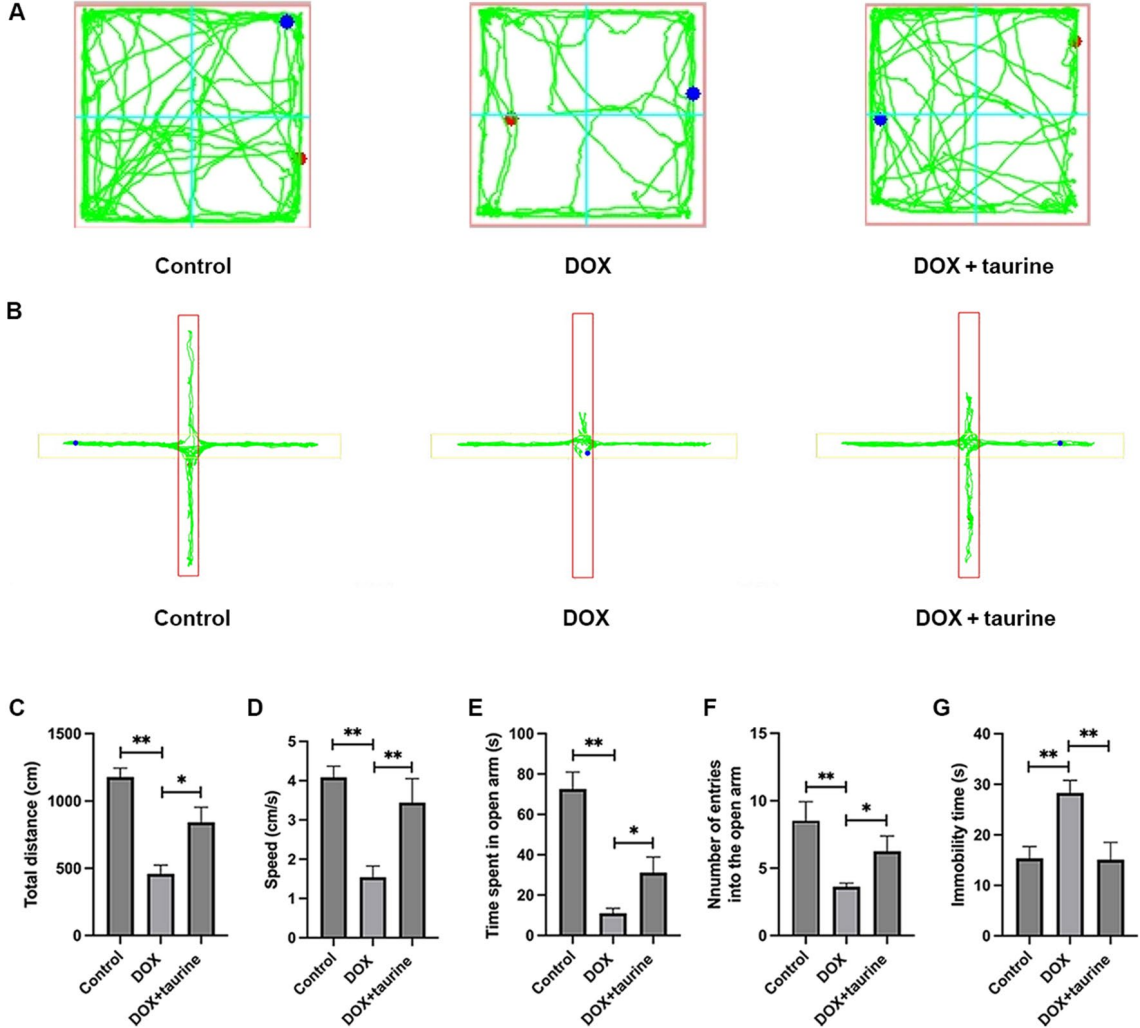
Effects of DOX and taurine on the behavior of mice. (**A**) Representative trajectory plots of mice in the OFT. (**B**) Representative trajectory plots of mice in the EPM. (**C**) Total distance traveled of mice in the OFT. (**D**) Average speed of mice in the OFT. (**E**) Time spent by mice in the open arm. (**F**) Number of entries into the open arm. (**G**) Immobility time of mice in the FST. Experimental data are expressed as mean ± SEM. *P < 0.05, **P < 0.01.

The effects of DOX and taurine on the behaviour of OFT are shown in Fig. 2A,C,D. The results showed that the total distance travelled and speed were significantly reduced in the DOX group compared to the control group, and pretreatment with taurine significantly altered this behaviour. This result illustrates that when faced with a new environment, the mice in the DOX group showed reduced autonomous and exploratory behaviours compared to the control group and may be in a state of stress and anxiety. In contrast, compared to the mice in the DOX group, the mice treated with taurine showed an increase in both moving distance and speed. They also were more active. The EPM test results are shown in Fig. 2B,E,F. Mice in the DOX group entered the open arm less frequently and spent significantly less time in the open arm compared to the control group, implying that mice in the DOX group may be in an anxious state. In contrast, taurine pretreatment altered this phenomenon, suggesting that taurine pretreatment alleviated DOX-induced anxiety behaviour. Furthermore, the FST results showed that the immobility time of the DOX group increased significantly compared to that of the control group. At the same time, taurine pretreatment reduced the immobility time of the DOX group, as shown in Fig. 2G. This suggests that mice in the DOX group had a prolonged state of despair during the FST compared to the control and that taurine treatment significantly reduced the duration of despair in the mice. Taken together, these experiments indicate that taurine treatment has a protective effect on DOX-induced depressive-like behaviour.

### Taurine treatment induced transcriptional changes

**Figure 3:**
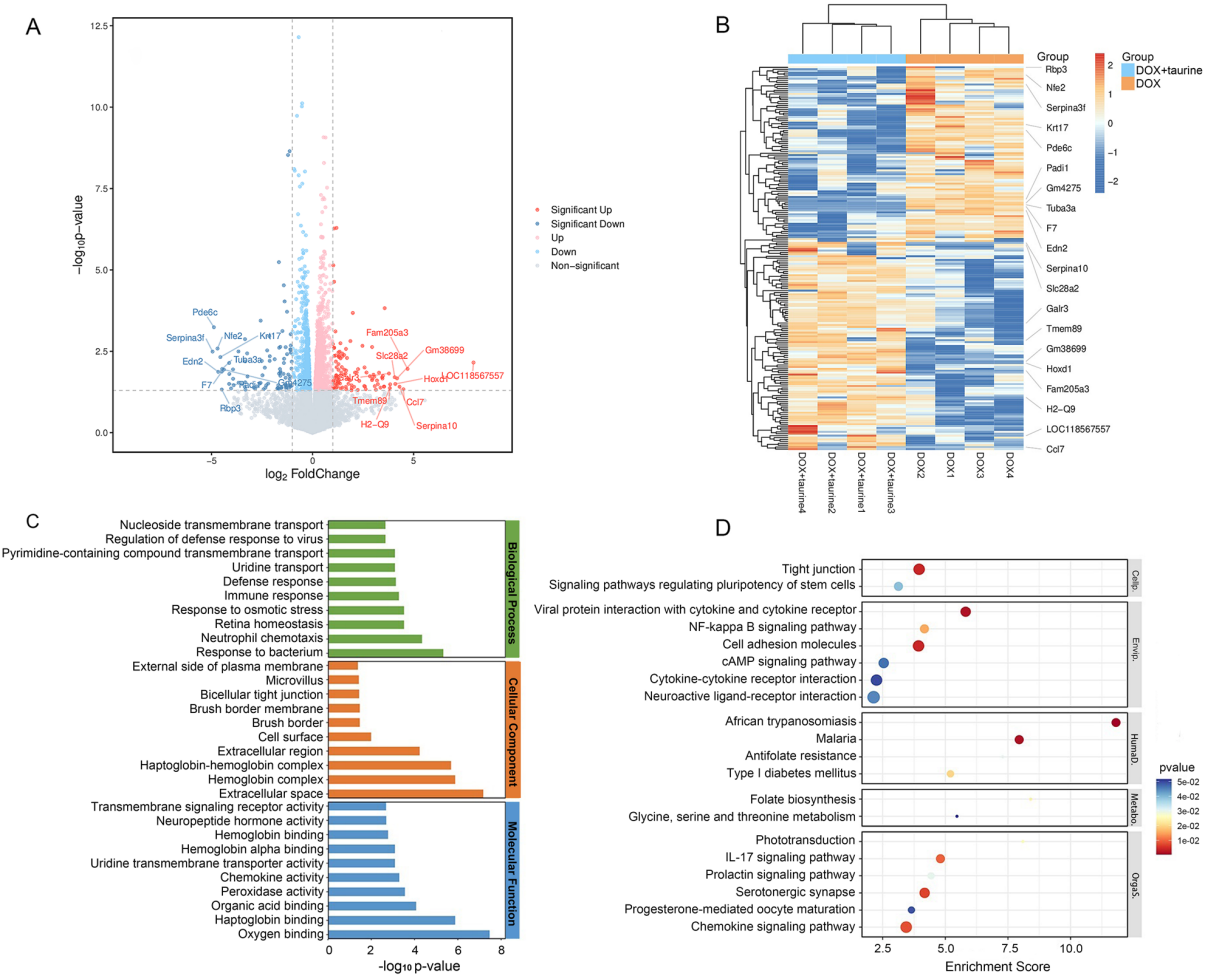
(**A**) Volcano plot of differentially expressed genes for DOX + taurine versus DOX. (**B**) Heatmap of hierarchical clustering analysis for DOX + taurine versus DOX. (**C**) GO enrichment classification histogram for the DOX + taurine versus DOX. (**D**) KEGG pathway analysis for the DOX + taurine versus DOX in transcriptomics.

Transcriptomics was used to analyse changes in the hippocampus at the gene level, using |log2FC| ≥ 1 and *P* < 0.05 as strict criteria for identifying differentially expressed genes. The results showed that 179 genes were significantly different in the DOX + taurine group compared to the DOX group, of which 109 showed upregulation and 70 showed downregulation. Specific information on the differentially expressed genes is shown in Supplementary Table 1. The volcano plot (Fig. 3A) and heatmap (Fig. 3B) visually demonstrate the differences in gene expression between groups. Samples were divided into two distinct clusters, indicating significant differences in the expression of genes in the different groups. Subsequently, GO enrichment and KEGG pathway analyses were performed on the differentially expressed genes. The histogram of GO enrichment classification in Fig. 3C shows the top 10 terms with the smallest *P* values among biological processes, cellular components, and molecular functions for display. The results showed that the differentially expressed genes played an important role in the extracellular space. Furthermore, the differentially expressed genes between the DOX and DOX + taurine groups were found to be involved in neutrophil chemotaxis, immune response, peroxidase activity, and chemokine activity. Specific information on the GO enrichment analysis is detailed in Supplementary Table 2. As shown in Fig. 3D, the results of the KEGG pathway analysis indicated that the differentially expressed genes in the DOX + taurine group were involved in multiple pathways, such as the NF-κB signalling pathway, the IL-17 signalling pathway, and the serotonergic synapse. Specific information on the KEGG pathway analysis is detailed in Supplementary Table 3.

### Taurine treatment induced metabolic changes

**Figure 4:**
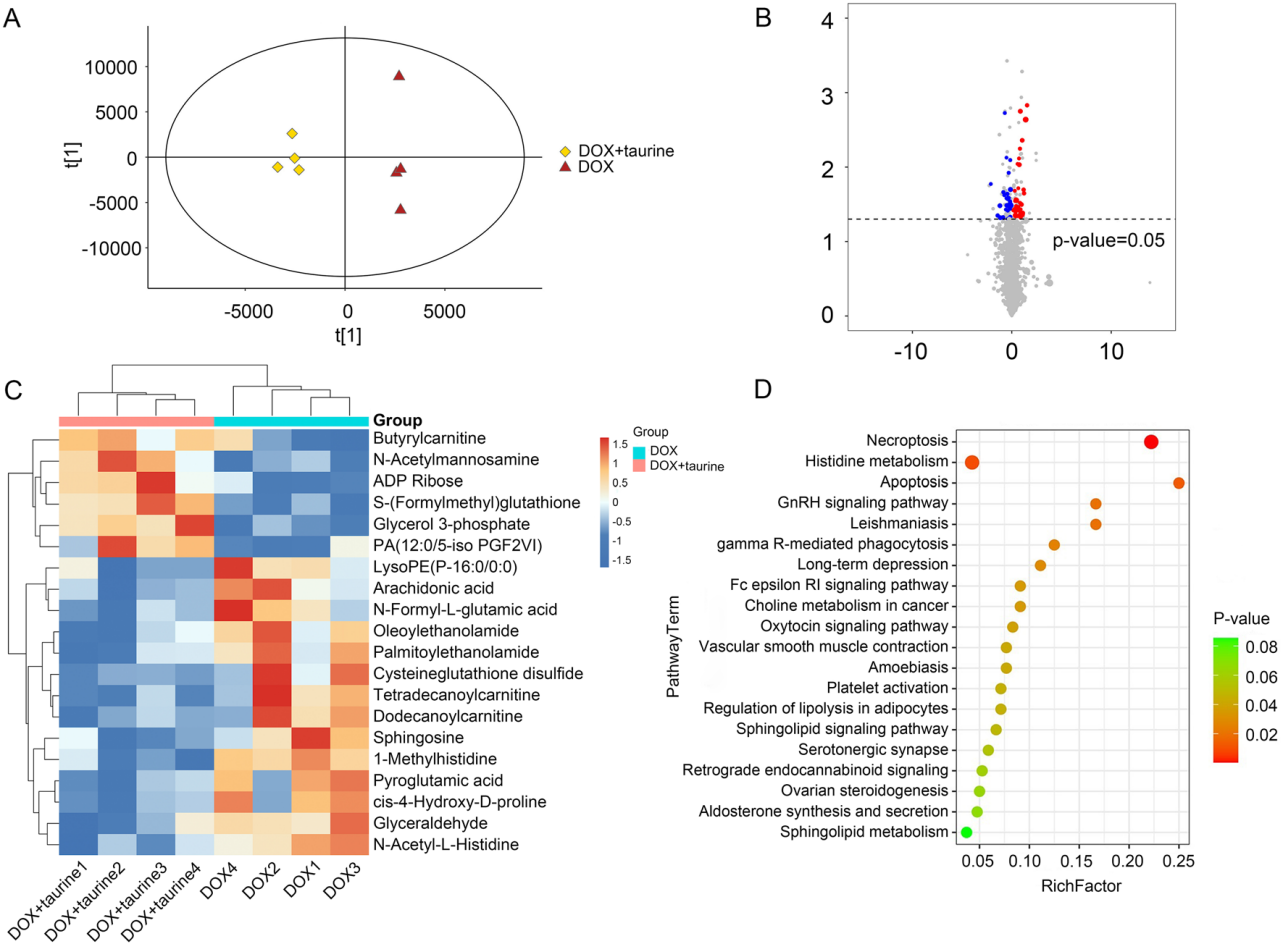
(**A**) Score scatter plot of the OPLS-DA model for the DOX + taurine versus DOX. (**B**) Volcano map of differentially expressed metabolites for the DOX + taurine versus DOX. (**C**) Heatmap of metabolites for the DOX + taurine versus DOX. (**D**) KEGG pathway analysis for the DOX + taurine versus DOX in metabolomics.

Metabolomic analysis was performed to assess the possible antidepressant effects of taurine at the metabolic level. Representative chromatograms from all three groups are shown in “Supplementary Figure”. The OPLS-DA showed that samples within the groups were clustered together. Samples between groups showed a clear separation, indicating significant differences between the two groups and low variability within the groups (Figs. 4A). VIP > 1 and *P* < 0.05 were used as strict criteria for screening metabolites with significant differences. Compared to the DOX group, 51 metabolites with significant differences were screened in the DOX + taurine group, of which 35 were upregulated and 28 were downregulated. Specific information on metabolites is detailed in Supplementary Table 4. The volcano plots provide information on the overall distribution of differentially expressed metabolites. Figure 4B shows the variation in metabolite expression in the DOX and DOX + taurine groups. Heatmaps can more intuitively reflect metabolite clustering expression patterns. Only some important metabolites are shown in Fig. 4C. These important differentially abundant metabolites showed similar expression trends within the groups and opposite expression trends between the groups, indicating suitable reproducibility of the samples in each group. Subsequently, the KEGG pathway library was used to analyse the metabolic pathways that these differentially abundant metabolites are involved in, and the results are shown in Fig. 4D. Multiple metabolic pathways, including apoptosis, long-term depression, and the serotonergic synapse, were screened in the DOX + taurine group. Specific information on the enriched metabolic pathways is presented in Supplementary Table 5.

### Comprehensive analysis of metabolomics and transcriptomics

**Figure 5:**
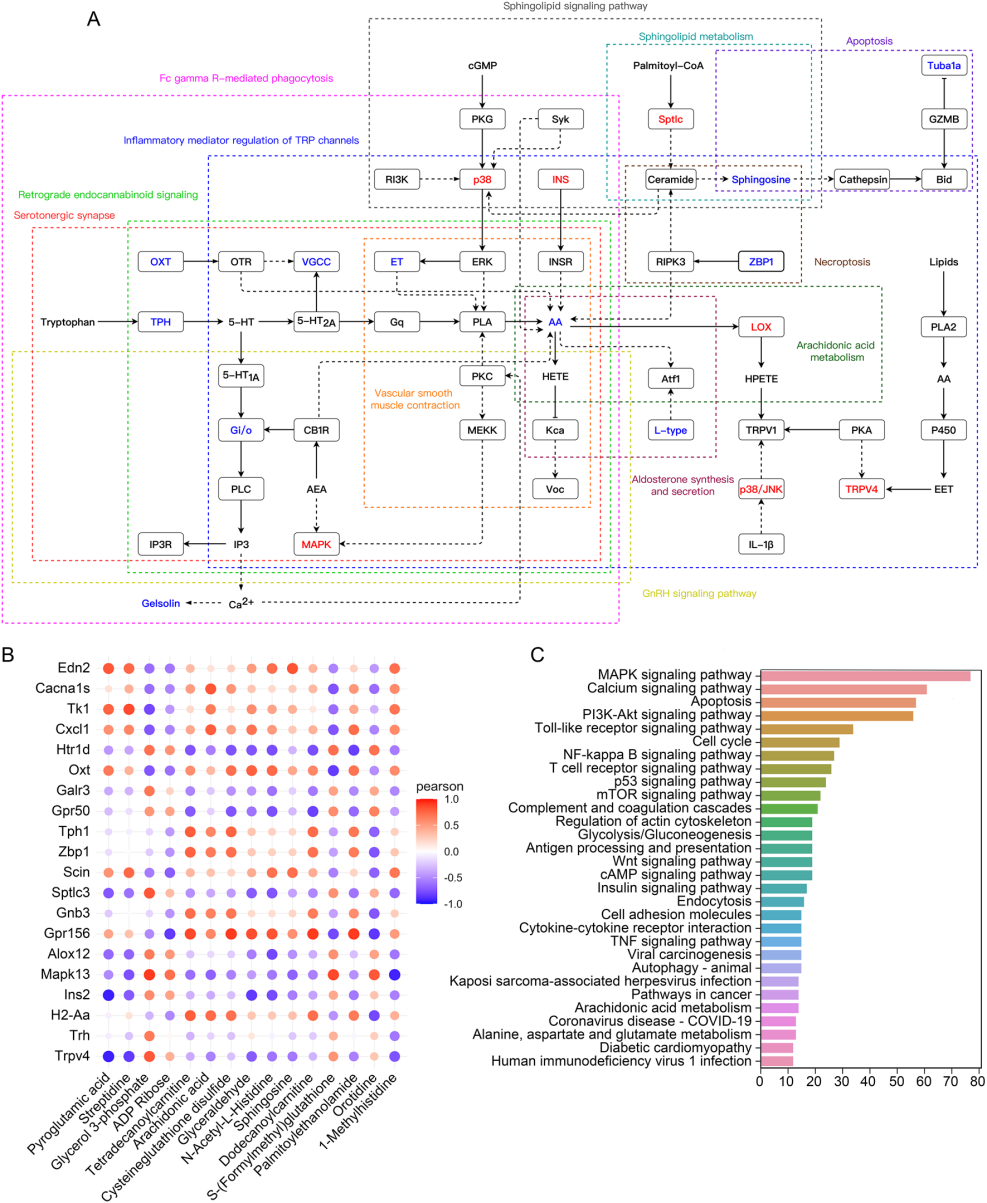
(**A**) Some critical pathways in which differential genes and differential metabolites are jointly involved. (Red represents upregulation, and blue represents downregulation) (**B**) Correlation between differential genes and differential metabolites. (**C**) Statistical results of KEGG pathway association elements.

Based on the above analysis, the KEGG pathway database was used to explore the pathways in which differential genes and differentially abundant metabolites were jointly involved, and the results showed that the DOX and DOX + taurine groups were involved in 21 metabolic pathways. Figure 5A shows some key metabolic pathways, including inflammatory mediator regulation of TRP channels, vascular smooth muscle contraction, arachidonic acid metabolism, and serotonergic synapses. Specific information on the enriched pathways is presented in Supplementary Table 6. In addition, the Pearson correlation algorithm was used to analyse the correlation between differential genes and metabolites. Figure 5B shows the correlation between some of the metabolites and genes. To analyse the interaction between transcriptomics and metabolomics more systematically, the upstream and downstream interactions of several pathways were further analysed based on the KGML file. The pathways with the top 30 counts were selected for visualization of the number of associated elements. As shown in Fig. 5C, the horizontal coordinates represent the number of elements associated with a pathway, and the vertical coordinates represent the names of the different pathways. The results show that the MAPK signalling pathway had the largest number of related elements.

## Discussion

We found that taurine exerts a significant protective effect against DOX-induced depression and subsequently evaluated the depression-like behaviour of mice and the possible antidepressant mechanism of taurine. Behavioural experiments showed that taurine pretreatment alleviated DOX-induced depression, manifested mainly by increasing the total travel time of mice in the OFT and the time spent in the open arm during the EMP test and reducing the immobility time in the OFT. Subsequently, the possible antidepressant effect of taurine was analysed using an integrated metabolomics and transcriptomics approach. A total of 179 differentially expressed genes and 51 metabolites with significant differences were screened in the DOX + taurine group. Then, a comprehensive analysis showed that differentially expressed genes and metabolites were jointly involved in 21 pathways, such as inflammatory mediator regulation of TRP channels, arachidonic acid metabolism, and serotonergic synapses. Abnormalities in these pathways have been strongly associated with the development of depression.

### The antidepressant effect of taurine is related to arachidonic acid

Combined transcriptomic and metabolomic analyses showed that differential genes and differentially abundant metabolites were jointly involved in 21 pathways, most of which involved arachidonic acid (AA). The results suggest that AA is a vital compound and may play an essential role in the antidepressant effect of taurine. AA is one of the most abundant fatty acids in the brain and has been closely linked to the regulation of inflammation^19^. AA has multiple metabolic pathways. Cytochrome P450, cyclooxygenase, and lipoxygenase (LOX) convert AA into eicosanoid mediators, including thromboxanes, prostaglandins, and leukotrienes, which are regulators of the inflammatory process^20^. In the present study, AA was significantly reduced in the DOX + taurine group, while Alox12, a gene encoding a member of the LOX family of proteins, was significantly upregulated. This finding suggests that taurine may regulate AA metabolism in response to DOX-induced depression via the LOX family.

AA is also involved in inflammatory mediator regulation of TRP channels. AA can be converted to HPETE by enzymes, which in turn are engaged in the regulation of Trpv1 channels. In addition, the Trpv4 channel may also be affected, and Trpv4 gene expression was also changed in the DOX + taurine group. TRPV4, as a Ca^2+^ channel, can mediate the influx of extracellular Ca^2+^, leading to changes in signalling and transcription. The formation of AA through phospholipase A2 (PLA2) and its metabolism by cytochrome P450 enzymes to 5,6-EET can activate TRPV4 and cause Ca^2+^ influx. This process is also the primary mechanism of endothelium-derived vasodilatation^21^. Therefore, the observed changes in the vascular smooth muscle contraction pathway may also be related to arachidonic acid abnormalities and TRPV4 activation^22^. Activation of TRPV4 mediates neutrophil adhesion and chemotaxis and generates reactive oxygen species, which upregulates the expression of proinflammatory cytokines, leading to neuroinflammation^23^. Inhibition of the TRPV4 pathway can alleviate inflammation-induced injury^24^. Based on the above analysis, the antidepressant effect of taurine may be related to changes in AA metabolism and TRPV channels. Taurine may respond to DOX-induced depression by modulating 5-HT.

### Taurine may alleviate DOX-induced depression by modulating 5-HT

Abnormalities of the serotonergic synapse were observed in the DOX-induced depression group, and taurine may alleviate depression-like behaviour by modulating the serotonergic synapse. Serotonin, also known as 5-hydroxytryptamine (5-HT), is synthesized from tryptophan in two steps. First, tryptophan hydroxylase (TPH) converts tryptophan to 5-hydroxy-L-tryptophan (5-HTP), which is the first and rate-limiting step in serotonin synthesis, and then 5-HTP is converted to serotonin by an enzyme^25^. In this study, Tph1, a related gene encoding TPH, was downregulated in the DOX + taurine group, indicating that the synthesis of 5-HT may be affected. In addition, serotonergic dysfunction is known to play a central role in regulating depression, and many antidepressants have been studied^26^. Serotonergic dysfunction has been reported to be closely associated with changes in 5-HT receptors, especially 5-HT_1A_ receptors^27^. 5-HT_1A_ receptors are among the most abundant serotonin receptors in the brain. The main effects of 5-HT binding to 5-HT_1A_ receptors are activating hyperpolarized K^+^ channels and inhibiting adenylate cyclase. These reactions are mediated by G proteins (Gi/Go) and cause membrane hyperpolarization and a decrease in Ca^2+^ influx, which finally reduces the neural discharge rate^25^. Although the exact mechanism is unclear, animal studies have shown that both the stimulation and blockade of 5-HT_1A_ receptors can cause or accelerate antidepressant effects^28^. In the present study, the expression of Htr1d, a gene associated with 5-HT_1A_, was upregulated in the DOX + taurine group. Gnb3, a gene associated with L-type calcium channels and Gi/Go, was also altered. This result suggests that the antidepressant effect of taurine may also be related to 5-HT_1A_ and Gi/Go.

In addition to 5-HT_1A_ receptors, 5-HT_2A_ receptors also play an important role in serotonergic synapses. 5-HT_2A_ receptors can affect L-type voltage-dependent calcium channels (L-VDCCs), which can regulate Ca^2+^ concentrations. The balance of Ca^2+^ is essential for maintaining intracellular balance^29^. Microglial activation by lipopolysaccharide (LPS) has been reported to increase Ca^2+^ influx, while the addition of a Ca^2+^ chelator reduced LPS-induced reactive oxygen species and proinflammatory cytokines released from microglia, suggesting that Ca^2+^ homeostasis and inflammation are closely related^30^. L-VDCC antagonists can also inhibit the expression of proinflammatory cytokines, leading to anti-inflammatory effects^31^. In the present study, cacna1s was one of the genes encoding L-VDCC, and the expression of cacna1s was downregulated in the DOX + taurine group. Therefore, taurine may protect against DOX-induced inflammation by regulating L-VDCC. In addition, the 5-HT_2A_ receptor coupled to the G protein of the Gq/11 type also regulates AA metabolism by affecting PLA2. Alterations in AA and Alox12 in the serotonergic synapse pathway were also found. Based on the above analysis, these findings suggest that taurine may alleviate DOX-induced depression by modulating serotonergic synapses and 5-HT.

## Conclusions

In summary, taurine pretreatment alleviated DOX-induced depressive behaviour. Taurine pretreatment increased the total travel time and speed of mice in the OFT, increased the number of entries into the open arm and the time spent in the open arm, and decreased the immobility time in the OFT. In addition, the results of this assay provide a basis for the antidepressant effects of taurine. The multiple pathways detected, such as serotonergic synapses and the inflammatory mediator regulation of TRP channels, are critical regulatory pathways that may be related to depression and antidepressant effects. The present study clarifies that taurine exerts a protective effect on DOX-induced depression and provides a preliminary exploration of the molecular pathways through which taurine may exert its effects. However, in-depth explorations are necessary to thoroughly understand the role of taurine in depression.

### Supplementary Information

Supplementary Information 1.

Supplementary Figure 1.

Supplementary Tables.

## Data Availability

The data presented in this study are available upon request from the corresponding author.

## Abbreviations

Dox: Doxorubicin
OFT: Open field test
EPM: Elevated plus maze test
FST: Forced swim test
AA: Arachidonic acid
LOX: Lipoxygenase
PLA2: Phospholipase A2
5-HT: 5-Hydroxytryptamine
5-HTP: 5-Hydroxy-L-tryptophan
L-VDCC: L-type voltage-dependent calcium channel
LPS: Lipopolysaccharide
