# Supplementary material for: Integrating transcriptomics and metabolomics to elucidate the mechanism by which taurine protects against DOX-induced depression
Source: Sci Rep. 2024 Feb 1;14:2686. doi: 10.1038/s41598-023-51138-5 (PMC10834502; doi:10.1038/s41598-023-51138-5)
Supplement: Supplementary file 1 — Supplementary Information 1. [file 41598_2023_51138_MOESM1_ESM.docx]

Supplementary Figure. Representative chromatograms from all three groups. (A) Control group, (B) DOX group, and (C) DOX+taurine group.
