# Supplementary figures and images for: Integrating transcriptomics and metabolomics to elucidate the mechanism by which taurine protects against DOX-induced depression
Source: Sci Rep. 2024 Feb 1;14:2686. doi: 10.1038/s41598-023-51138-5 (PMC10834502; doi:10.1038/s41598-023-51138-5)

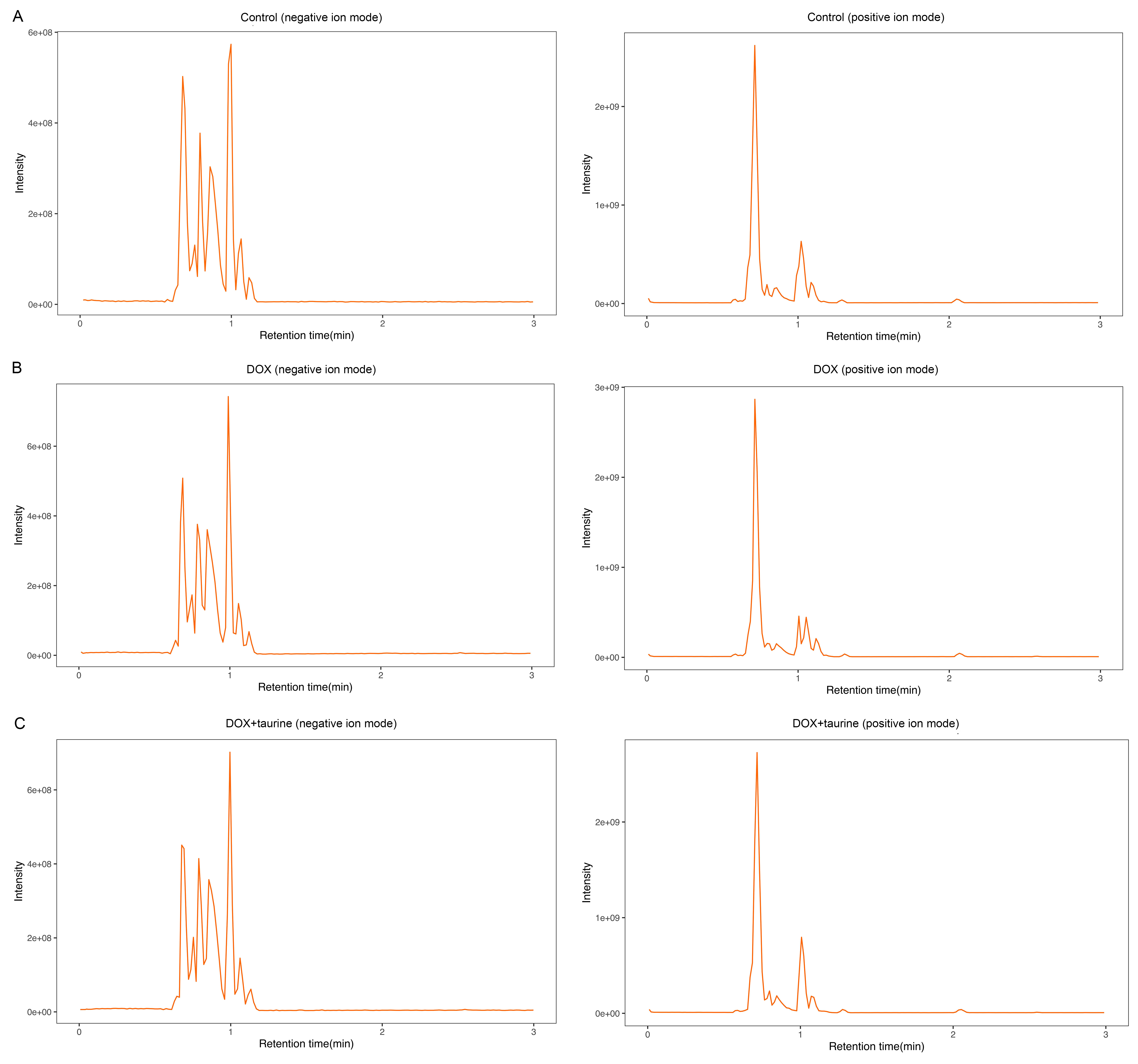

Supplement: Supplementary file 2 — Supplementary Figure 1. [file 41598_2023_51138_MOESM2_ESM.jpg]
